# Intra-Tumoral CD8+ T-Cell Infiltration and PD-L1 Positivity in Homologous Recombination Deficient Pancreatic Ductal Adenocarcinoma

**DOI:** 10.3389/fonc.2022.860767

**Published:** 2022-04-25

**Authors:** Bryn Golesworthy, Yifan Wang, Amanda Tanti, Alain Pacis, Joan Miguel Romero, Adeline Cuggia, Celine Domecq, Guillaume Bourdel, Robert E. Denroche, Gun Ho Jang, Robert C. Grant, Ayelet Borgida, Barbara T. Grünwald, Anna Dodd, Julie M. Wilson, Guillaume Bourque, Grainne M. O’Kane, Sandra E. Fischer, Chelsea Maedler Kron, Pierre-Olivier Fiset, Atilla Omeroglu, William D. Foulkes, Steven Gallinger, Marie-Christine Guiot, Zu-Hua Gao, George Zogopoulos

**Affiliations:** ^1^ The Research Institute of the McGill University Health Centre, Montreal, QC, Canada; ^2^ The Rosalind and Morris Goodman Cancer Institute of McGill University, Montreal, QC, Canada; ^3^ The Department of Surgery, McGill University, Montreal, QC, Canada; ^4^ The McGill Genome Center and Canadian Centre for Computational Genomics (C3G), Montreal, QC, Canada; ^5^ The Ontario Institute for Cancer Research, Toronto, ON, Canada; ^6^ The Lunenfeld Tanenbaum Research Institute, Mount Sinai Hospital, Toronto, ON, Canada; ^7^ The Wallace McCain Centre for Pancreatic Cancer, Princess Margaret Cancer Centre, University Health Network, Toronto, ON, Canada; ^8^ The Department of Human Genetics, McGill University, Montreal, QC, Canada; ^9^ The Division of Pathology, University Health Network, Toronto, ON, Canada; ^10^ The Department of Pathology, McGill University, Montreal, QC, Canada

**Keywords:** pancreatic cancer, tumor microenvironment, T-cell inflammation, immunotherapy, PD-L1

## Abstract

The immune contexture of pancreatic ductal adenocarcinoma (PDAC) is generally immunosuppressive. A role for immune checkpoint inhibitors (ICIs) in PDAC has only been demonstrated for the rare and hypermutated mismatch repair (MMR) deficient (MMR-d) subtype. Homologous recombination repair (HR) deficient (HR-d) PDAC is more prevalent and may encompass up to 20% of PDAC. Its genomic instability may promote a T-cell mediated anti-tumor response with therapeutic sensitivity to ICIs. To investigate the immunogenicity of HR-d PDAC, we used multiplex immunohistochemistry (IHC) to compare the density and spatial distribution of CD8+ cytotoxic T-cells, FOXP3+ regulatory T-cells (Tregs), and CD68+ tumor-associated macrophages (TAMs) in HR-d *versus* HR/MMR-intact PDAC. We also evaluated the IHC positivity of programmed death-ligand 1 (PD-L1) across the subgroups. 192 tumors were evaluated and classified as HR/MMR-intact (n=166), HR-d (n=25) or MMR-d (n=1) based on germline testing and tumor molecular hallmarks. Intra-tumoral CD8+ T-cell infiltration was higher in HR-d *versus* HR/MMR-intact PDAC (p<0.0001), while CD8+ T-cell densities in the peri-tumoral and stromal regions were similar in both groups. HR-d PDAC also displayed increased intra-tumoral FOXP3+ Tregs (p=0.049) and had a higher CD8+:FOXP3+ ratio (p=0.023). CD68+ TAM expression was similar in HR-d and HR/MMR-intact PDAC. Finally, 6 of the 25 HR-d cases showed a PD-L1 Combined Positive Score of >=1, whereas none of the HR/MMR-intact cases met this threshold (p<0.00001). These results provide immunohistochemical evidence for intra-tumoral CD8+ T-cell enrichment and PD-L1 positivity in HR-d PDAC, suggesting that HR-d PDAC may be amenable to ICI treatment strategies.

## Introduction

Pancreatic ductal adenocarcinoma (PDAC) is the seventh leading cause of cancer death worldwide and its incidence is rising yearly (1). It is one of the most lethal malignancies, with a 5-year survival rate of less than 10% (1). Nearly 80% of patients are diagnosed with incurable locally advanced or metastatic disease and are treated with systemic chemotherapy (2). However, the effectiveness of empiric chemotherapeutic regimens, such as FOLFIRINOX (5-fluorouracil, leucovorin, irinotecan and oxaliplatin) and gemcitabine plus nab-paclitaxel, remains poor (2). The 20% of patients who undergo curative-intent resection and receive adjuvant therapy reach a median overall survival of only 22.8 to 54.4 months, which highlights the systemic behavior of PDAC even at its earliest stage (3–5).

While empiric chemotherapy regimens remain the backbone of systemic treatment for PDAC, these treatment strategies have not led to marked improvements in survival [2]. Thus, guided by biomarker-driven treatment breakthroughs in other difficult-to-treat cancers and facilitated by the identification of PDAC molecular subtypes, precision medicine and immunotherapy strategies have emerged. The most characterized molecular PDAC subtypes are based on 1) genomic tumor alterations driven either by germline predisposition or somatic oncogenic aberrations and 2) transcriptomic expression patterns (6). Associations of these subtypes with PDAC progression and treatment responses have demonstrated their potential clinical value (7–9).

Homologous recombination repair (HR) deficiency (HR-d) PDAC is an actionable molecular subtype. It is primarily driven by germline mutations in *BRCA2*, *BRCA1* and *PALB2*, which are present in 5-10% of incident PDAC cases (2, 10–13). In addition, 7-10% of PDAC cases without germline mutations in HR-associated genes harbor molecular hallmarks of HR-d driven by somatic or epigenetic events (14). HR-d PDAC is sensitive to platinum-based cytotoxic regimens and poly(ADP-ribose) polymerase (PARP) inhibitors, which exploit the intrinsic deficiency of HR-d tumor cells to repair DNA double strand breaks with high-fidelity (15). In addition, the genomic instability of HR-d tumors, characterized by specific genomic alterations that include deletions with flanking microhomology and the COSMIC Signature 3 pattern of base-substitution mutations, may lead to increased neoantigens and a T cell-mediated anti-tumor response with therapeutic sensitivity to immune checkpoint inhibitors (ICIs) (16, 17). Durable treatment responses to ICIs have been demonstrated in cancers with T cell-inflamed phenotypes, but the ability of HR-d PDAC to induce anti-tumor immunity has not been established (18). Moreover, the PDAC tumor microenvironment is generally immunosuppressive and a role for ICIs in PDAC has only been demonstrated for the rare and hypermutated mismatch repair (MMR) deficient (MMR-d) subtype, which exhibits inherent immunogenicity (19, 20). Thus, to assess the potential actionability of HR-d PDAC using ICIs, we characterized the spatial distribution of immune cells relative to tumor cells in HR-d *versus* HR/MMR-intact PDAC.

## Materials and Methodology

### Patient Cohorts

Tumor specimens from patients with pathological diagnoses of PDAC from two case series were evaluated. The first series included 141 PDAC cases from the Quebec Pancreas Cancer Study [QPCS, NCT04104230 (21)]. This series consisted of 130 consecutive patients enrolled in the QPCS between April 2012 and September 2018, with available primary resected PDAC specimens for construction of a tissue microarray (TMA). To compensate for the lower incidence of HR-d and MMR-d compared to HR/MMR-intact PDAC, we also included cases with germline mutations in HR or MMR genes (n=11) that were enrolled in the QPCS following construction of the TMA. These included primary (n=8) and metastatic (n=3) PDAC specimens from cases enrolled between October 2018 and December 2020. The second series consisted of 115 cases from the PanCuRx Translational Research Initiative, which were represented on a previously constructed TMA (22). Patient demographics, clinical characteristics and survival outcomes for both series were extracted from prospectively maintained study databases. Overall survival was calculated from the date of radiological diagnosis until death or censor date. Clinical staging was based on the 8th edition of the American Joint Committee on Cancer.

### Construction of Tissue Microarrays

TMAs for the QPCS cases were created using an automated tissue microarrayer (TMA Grand Master, 3DHistech, RRID: SCR_021257). Representative tumor regions were identified on hematoxylin and eosin (H&E) stained slides by a board-certified pathologist (A.O., Z.H.G.) and marked on its corresponding formalin-fixed and paraffin-embedded (FFPE) block. Each case (n=130) was represented on a TMA by three 1.5-mm tumor cores. The PanCuRx TMA was previously constructed and included 115 cases that were each represented by at least two 1.5-mm cores (22).

### Immunostaining and Spatial Analysis of Tumor Infiltrating Immune Cells

TMA and individual patient blocks were cut at a 4-micrometer (μm) thickness for immunohistochemistry (IHC). Multiplex chromogenic IHC was performed using the Discovery Ultra Autostaining Platform (Ventana Medical Systems, RRID: SCR_021254), with chromogenic detection kits from Ventana Medical Systems (No. 760-247, teal; No. 760-229, purple; No. 760-500, DAB, RRID: AB_2753116; No. 760-250, yellow; No. 760-271, green). QPCS and PanCuRx slides were stained for CD8 (Ventana, 790-4460, RRID: AB_2335985), Pan-cytokeratin (PanCK; Ventana, 760-2135, RRID: AB_2810237), Forkhead box P3 (FOXP3; 1:200, Abcam, ab20034, RRID: AB_445284), and programmed death-ligand 1 (PD-L1; E1L3N clone, 1:100, Cell Signaling, 13684S, RRID: AB_2687655) in combination with the DISCOVERY Amp HQ kit (Ventana, 760-4602). The QPCS series was also stained for CD68 (1:100, Abcam, ab125212, RRID: AB_10975465). Staining specificity was confirmed by board-certified pathologists (M.C.G., P-O.F.).

Immunostained slides were scanned using the Aperio AT2 ScanScope (Leica Biosystems, RRID: SCR_021256) at a 20x magnification. We trained a classifier algorithm on the HALO Image Analysis software (Indica Labs; RRID: SCR_018350) to use PanCK staining for assignment of tumor cell clusters. Regions of necrosis, blood vessels, acinar cells and islet cells were excluded. Tumor and immune cells were counted using the Multiplex-IHC v.3.0.4 package on HALO and averaged across replicate tumor cores for each case. Immune cell densities were calculated by normalizing immune cell counts to the total area of tumor clusters (mm^2^). For log_10_ transformation, cases with immune cell counts of zero were assigned a value corresponding to 90% of the lowest non-zero immune count in the cases evaluated.

**Figure 1:**
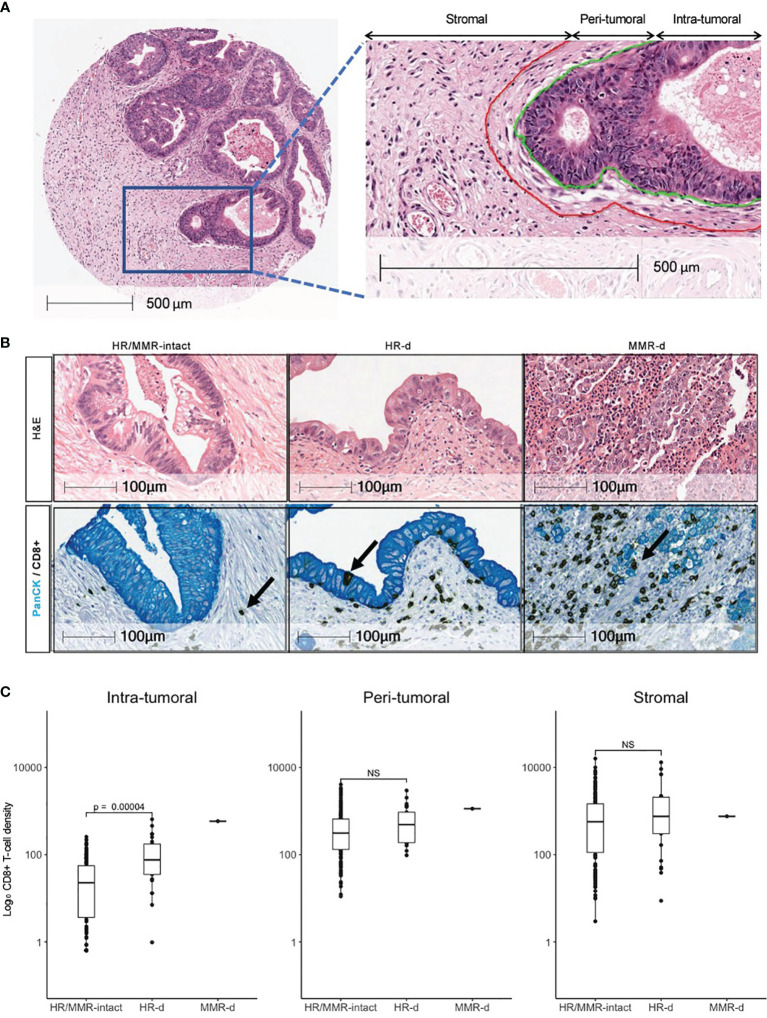
Distribution of CD8+ T-cells in PDAC. **(A)** Definitions of intra- tumoral, peri- tumoral and stromal regions. **(B)** Representative H&E images for HR/MMR-intact, HR-d and MMR-d PDAC with corresponding immunostaining for CD8 (brown) and Pan-cytokeratin (PanCK, teal). Black arrows show examples of CD8+ staining. **(C)** Comparison of CD8+ T-cell densities in HR/MMR-intact *versus* HR-d PDAC across the three tumor regions. The MMR-d case is shown as a reference for an immunogenic PDAC. NS, not significant.

To distinguish between immune cells infiltrating the tumor *versus* those encroaching the tumor perimeter, immune cells were classified as intra-tumoral if they were located within 10 μm from the edge of a tumor cell (**Figure 1A**). Immune cells were considered peri-tumoral if they were located between 10 μm and 50 μm from the tumor cell cluster perimeter. Immune cells that were more than 50 μm away from a tumor cell cluster perimeter were categorized as stromal. The CD8+:FOXP3+ ratio was calculated by dividing the total number of CD8+ cytotoxic T-lymphocytes by the total number of FOXP3+ regulatory T-lymphocytes (Tregs) in each core, and averaging across tumor replicates. The Combined Positive Score (CPS) was calculated for each core as previously described (23) and averaged across tumor replicates. A CPS of ≥1 was considered positive. The HALO analysis for PD-L1 staining was validated with manual scoring by a board-certified pathologist (M.C.G.).

### Identification of HR-d and MMR-d Cases

Across the two series, 192 cases (QPCS n=114; PanCuRx n=78) were evaluable (**Table 1** and **Supplementary Table 1**). These cases were classified into HR/MMR-intact, HR-d or MMR-d subgroups and their immune tumor microenvironment was compared (**Supplementary Tables 2**, **3**). To identify HR-d cases in the QPCS series, we performed germline testing using lymphocyte DNA for *BRCA1*, *BRCA2*, and *PALB2* mutations. There were 52 cases previously tested by whole genome sequencing (WGS, n=11), whole exome sequencing (WES, n=1), or targeted sequencing using genes panels that included *BRCA1*, *BRCA2*, and *PALB2* (n=40, **Supplementary Table 4**) (13, 24, 25). The remaining 62 cases that had not undergone germline genetic testing were evaluated using the INVITAE Multi-Cancer gene panel (**Supplementary Table 4**). Germline mutations for the PanCuRx series were abstracted from previously reported WGS of lymphocyte DNA (22).

**Table 1:** Clinical characteristics of the 192 evaluable PDAC cases.

	HR/MMR-intact	HR-d	MMR-d
	(n=166)	(n=25)	(n=1)
**Age at diagnosis, mean ± SD**	66.3 ± 10.0	59.7 ± 11.7	55.0
**Gender, n (%)**			
Male	91 (54.8)	15 (60.0)	0 (0)
Female	75 (45.2)	10 (40.0)	1 (100)
**Stage at diagnosis, n (%)**			
Early Stage (I & II)	159 (95.8)	12 (48.0)	1 (100)
Late Stage (III & IV)	7 (4.2)	13 (52.0)	0 (0)
**Primary tumor resection specimens or biopsies, n (%)**			
Treated	26 (15.7)	9 (36.0)	0 (0)
Treatment Naïve	139 (83.7)	14 (56.0)	1 (100)
**Metastatic tumor biopsies, n (%)**			
Treated	0 (0)	1 (4.0)	0 (0)
Treatment Naïve	1 (0.6)	1 (4.0)	0 (0)

SD, standard deviation.

For cases that had undergone tumor whole genome sequencing (8, 17, 22), we calculated the HRDetect score using previously described methodology (26). Assignment of cases to the HR-d subgroup was based on the presence of a germline *BRCA1*, *BRCA2* or *PALB2* mutation and, if available, an HRDetect score of ≥0.9. If an HRDetect score was unavailable, cases with germline mutations were kept in the HR-d subgroup. Cases that carried a germline mutation, but did not meet the HRDetect threshold, were considered HR-intact. An HRDetect score of >0.7 for a low tumor cellularity case (303.001) was accepted to signify HD-d. In the absence of a germline HR-gene mutation, cases that had an HRDetect score >0.9 and evidence of somatic HR-gene inactivation were classified as HR-d.

We surveyed the QPCS and PanCuRx case series for germline MMR-gene mutations to identify potential MMR-d cases (**Supplementary Tables 2, 3**). However, assignment to the MMR-d subgroup was based on IHC for MMR protein deficiency (i.e., MLH1, MSH2, MSH6, PMS2) or an MSIsensor score ≥20 (https://github.com/niu-lab/msisensor2). For the QPCS series, IHC for MLH1 (G168-15, Biocare Medical, RRID: AB_1059376), MSH2 (G219-1129, Cell Marque, RRID: AB_1160591), MSH6 (EPR3945, Abcam, RRID: AB_2144959) and PMS2 (EPR3947, Cell Marque) was performed using a BenchMark ULTRA IHC Staining Module (Roche Diagnostics) with the OptiView DAB IHC Detection Kit. IHC staining was analyzed using the ImageScope software. Cases were considered MMR-intact if tumor cells displayed nuclear staining of all 4 MMR proteins. Cases were classified as MMR-d if tumor cells had complete loss of nuclear staining in one or more MMR proteins, with retained nuclear staining in adjacent stroma. Absence of MMR protein expression on a TMA core was confirmed by staining a whole section slide. MMR-d classification for the PanCuRx case series was based on previously reported IHC for MLH1, MSH2, MSH6 and PMS2 or MSIsensor scores (19, 22). Cases were assigned to the HR/MMR-intact subgroup if they did not meet criteria for HR-d or MMR-d subclassification or if they could not be evaluated for tumor molecular hallmarks due to biospecimen availability.

### Statistical Analyses

All statistical analyses were performed using R Software (version 4.0.4, R Foundation for Statistical Computing). Continuous variables were expressed as mean ± standard deviation (SD), and differences were compared using the Wilcoxon test. Fisher’s Exact Test was used to compare the proportion of cases in the HR/MMR-intact *versus* HR-d groups meeting the PD-L1 CPS threshold of ≥1. Overall survival was estimated using the Kaplan–Meier method and compared between groups using a log-rank test.

### Study Approval

All participants provided written informed consent. The study was conducted in accordance with the principles of the Declaration of Helsinki. The McGill University and the McGill University Health Centre (MUHC) Institutional Review Boards (#A02-M118-11A, #2018-3171, #2018-4139) approved the QPCS study, and the Institutional Review Board of the University Health Network (#15-9596) provided approval for the PanCuRx case series.

## Results

### Clinical Characteristics

Across the QPCS and PanCuRx case series, 192 PDAC cases were characterized, which included 166, 25, and 1 HR/MMR-intact, HR-d, and MMR-d cases, respectively (**Table 1**). The germline mutations and tumor genomic features of the HR-d and MMR-d cases are summarized in **Table 2**, and their clinical characteristics at tumor biospecimen acquisitions are detailed in **Table 3**. The HR-d group demonstrated a longer median overall survival (OS; 29.1 months *versus* 19.9 months, p=0.0073; **Supplementary Figure 1**). Notably, the HR-d group included a greater number of patients diagnosed with stage III/IV disease compared to the HR/MMR-intact group (52.0% *versus* 4.2%, p<0.0001).

**Table 2:** Germline mutations and tumor genomic features of the HR-d and MMR-d cases.

Subgroup Classification	ID	Germline Mutation	Somatic (Tumor) Alteration ¶	HRDetect Score †	MSIsensor Score †	MMR IHC
**HR-d**	348.001	*BRCA1* c.2681_2682delAA				Intact
	1048.001	*BRCA1* c.1018C>T	*BRCA1* LOH	>0.999	1.76	
	PCSI_0476	*BRCA1* c.5319dupC	*BRCA1* deletion (chr17:41249032-chr17:56361777)	>0.999	2.05	
	70.001	*BRCA2* c.3398del5	*BRCA2* c.1794_1798del	>0.999	2.17	Intact
	99.001	*BRCA2* c.4691dupC				Intact
	392.001	*BRCA2* c.8677C>T	*BRCA2* c.2050C>T	>0.999	1.62	Intact
	543.001	*BRCA2* c.3545delTT				Intact
	908.001	*BRCA2* c.8297delC	*BRCA2* LOH	>0.999	0.17	Intact
	1024.001	*BRCA2* c.1805_1806insA	*BRCA2* LOH	>0.999	2.2	
	1183.001	*BRCA2* c.4284dup				Intact
	1195.001	*BRCA2* c.3170_3174del				Intact
	1227.001	*BRCA2* c.8537_8538del				Intact
	1235.001	*BRCA2* c.3170_3174del				n/a
	1337.001	*BRCA2* c.6275_6276del				Intact
	PCSI_0017	*BRCA2* c.5946delT	*BRCA2* LOH *	>0.999	2.44	
	PCSI_0048	*BRCA2* c.5946delT	*BRCA2* LOH	>0.999	0.96	Intact
	PCSI_0075	-	*BRCA2* c.5718_5719del, *BRCA2* c.6579A>G	>0.999	1.46	Intact
	PCSI_0142	*BRCA2* c.9435_9436delGT	*BRCA2* LOH	>0.999	1.74	Intact
	PCSI_0176	*BRCA2* c.3167_3170delAAAA	*BRCA2* LOH *	>0.999	1.14	
	PCSI_0218	*BRCA2* c.3167_3170delAAAA	*BRCA2* c.8910G>A	>0.999	0.73	Intact
	PCSI_0472	-	*BRCA2* c.5718_5719del, *BRCA2* c.316+1G>T	>0.999	2.32	
	PCSI_0477	*BRCA2* c.9097dupA	*BRCA2* LOH	>0.999	1.69	
	PCSI_0492	*BRCA2* c.4003G>T	*BRCA2* LOH	>0.999	2.80	
	303.001	*PALB2* c.2323C>T	*PALB2* c.2174C>G	0.742 §	1.36	Intact
	1099.001	*PALB2* Deletion (exon 11)				Intact
**MMR-d**	750.001	*MSH2* c.942+3A>T				MSH2 & MSH6 deficient

¶ Somatic alterations were ascertained by whole genome sequencing.

† Shown are available results for cases with tumor whole genome sequencing.

* Patient-derived tumor xenograft tissue was used for whole genome sequencing when the patient tumor sample was insufficient.

§ Low tumor cellularity following laser microdissection (30.1%), which may have resulted in uncalled structural events and an HRDetect score of 0.742.

- Indicates no germline mutation identified.

LOH, loss of heterozygosity. MMR IHC, immunohistochemistry for mismatch repair proteins.

n/a Indicates insufficient tissue for immunohistochemistry.

**Table 3:** Clinical characteristics of the HR-d and MMR-d cases at tissue acquisition.

Subgroup Classification	ID	Age at Diagnosis (years)	Sex	Stage	Chemotherapy Prior to Tissue Acquisition	Radiation Therapy Prior to Tissue Acquisition	Tissue Acquisition Procedure	Tissue Acquired
**HR-d**	348.001	77	M	II	No	No	Pancreaticoduodenectomy	Primary Tumor
	1048.001	64	M	IV	No	No	Percutaneous Biopsy	Primary Tumor
	PCSI_0476	42	M	IIA	**FFX**	No	Pancreaticoduodenectomy	Primary Tumor
	70.001	47	M	IV	**FFX**	No	Distal pancreatectomy + splenectomy + RFA of liver metastases	Primary Tumor
	99.001	46	M	III	**FFX, GC**	**Yes**	Pancreaticoduodenectomy + PV resection + SMA resection	Primary Tumor
	392.001	61	F	II	No	No	Pancreaticoduodenectomy	Primary Tumor
	543.001	75	M	IV	No	No	Percutaneous Biopsy	Liver Metastasis
	908.001	53	F	III	**FFX**	No	Pancreaticoduodenectomy	Primary Tumor
	1024.001	70	M	IV	No	No	Percutaneous Biopsy	Primary Tumor
	1183.001	57	F	III	No	No	Percutaneous Biopsy	Primary Tumor
	1195.001*	74	F	II	No	No	Percutaneous Biopsy	Primary Tumor
	1227.001	39	M	IV	No	No	Percutaneous Biopsy	Primary Tumor
	1235.001	60	F	III	No	No	Percutaneous Biopsy	Primary Tumor
	1337.001	62	F	III	No	No	Percutaneous Biopsy	Primary Tumor
	PCSI_0017	53	F	III	**GC**	No	Pancreaticoduodenectomy	Primary Tumor
	PCSI_0048	76	M	IB	No	No	Pancreaticoduodenectomy	Primary Tumor
	PCSI_0075	75	M	IIA	No	No	Distal pancreatectomy	Primary Tumor
	PCSI_0142	43	M	IIB	No	No	Pancreaticoduodenectomy	Primary Tumor
	PCSI_0176	56	F	IB	**GC**	**Yes**	Pancreaticoduodenectomy	Primary Tumor
	PCSI_0218	50	M	IIB	No	No	Pancreaticoduodenectomy	Primary Tumor
	PCSI_0472	75	M	IA	No	No	Pancreaticoduodenectomy	Primary Tumor
	PCSI_0477	63	M	IB	No	No	Pancreaticoduodenectomy	Primary Tumor
	PCSI_0492	66	F	III	**GC**	No	Pancreaticoduodenectomy	Primary Tumor
	303.001	56	M	III	**FFX**	**Yes**	Total pancreatectomy + PV resection + right hemicolectomy	Primary Tumor
	1099.001	52	F	III	**FFX**	**Yes**	Surgical exploration/metastatic peritoneal biopsy	Peritoneal Metastasis
**MMR-d**	750.001	55	M	II	No	No	Subtotal pancreatectomy + splenectomy	Primary Tumor

*Treated concrrently for for lung cancer and excluded from the overall survival analysis.

RFA, radiofrequency ablation; PV, portal vein; SMA, superior mesenteric artery.

FFX, FOLFIRINOX; GC, gemcitabine/cisplatin.

### Increased Intra-tumoral Density of CD8+ T-cells and FOXP3+ Tregs in HR-d PDAC

HR-d tumors had a higher CD8+ T-cell intra-tumoral density compared to the HR/MMR-intact group (131.1 ± 154.9 cells/mm^2^ *versus* 40.5 ± 50.9 cells/mm^2^, p<0.0001; **Figure 1**). However, there was no difference in CD8+ T-cell infiltration in the peri-tumoral or stromal regions between the two groups. The CD8+ T-cell landscape for the MMR-d case is shown in parallel as a reference for a PDAC microenvironment responsive to immunotherapy. Following resection of the primary and adjuvant therapy with gemcitabine/capecitabine, this patient (750.001) developed a mesenteric recurrence, which responded to pembrolizumab (**Supplementary Figure 2**).

**Figure 2:**
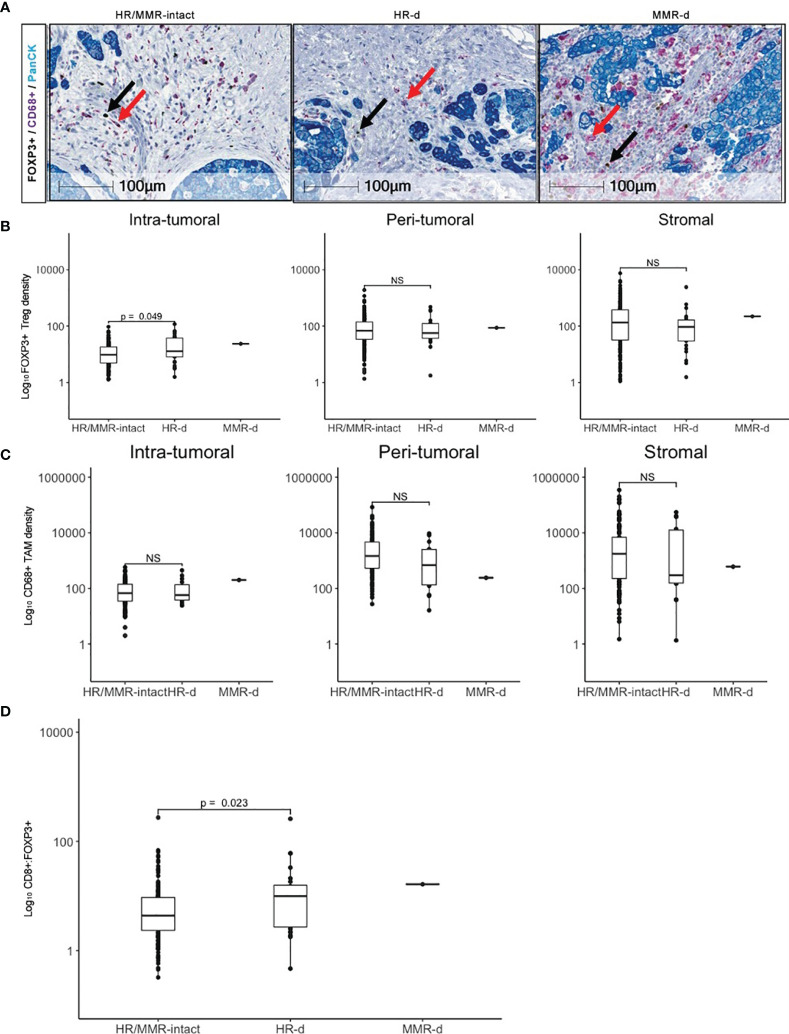
FOXP3+ Treg and CD68+ TAM infiltration in PDAC. **(A)** Representative FOXP3 (brown), CD68 (purple) and PanCK (teal) immunostaining for HR/MMR-intact, HR-d and MMR-d PDAC. Black and red arrows show examples of FOXP3+ and CD68+ staining, respectively. **(B, C)** Comparison of FOXP3+ Treg **(B)** and CD68+ TAM **(C)** densities in HR/MMR-intact *versus* HR-d PDAC across the intra-tumoral, peri- tumoral and stromal regions. **(D)** Comparison of overall CD8+:FOXP3+ ratios between HR/MMR-intact *versus* HR-d PDAC. The MMR-d case is shown as a reference. NS, not significant.

We subsequently evaluated the landscape of FOXP3+ Tregs and CD68+ tumor-associated macrophages (TAMs) in HR/MMR-intact *versus* HR-d tumors (**Figure 2**). FOXP3+ Tregs density was higher in the intra-tumoral region of HR-d *versus* HR/MMR-intact tumors (25.5 ± 27.3 cells/mm^2^ *versus* 13.6 ± 13.4 cells/mm^2^, p=0.049), while there was no difference in FOXP3+ staining in the peri-tumoral or stromal regions between the two groups. Moreover, the ratio of CD8+ to FOXP3+ cells was higher in HR-d *versus* HR/MMR-intact cases (23.9 ± 52.7 *versus* 9.8 ± 23.8, p=0.023). Similarly, the CD8+ to FOXP3+ ratio in the MMR-d tumor was also elevated. CD68+ TAM population densities were comparable across the three spatial compartments in HR-d and HR/MMR-intact PDAC.

### Increased PD-L1 Expression in HR-d PDAC

**Figure 3:**
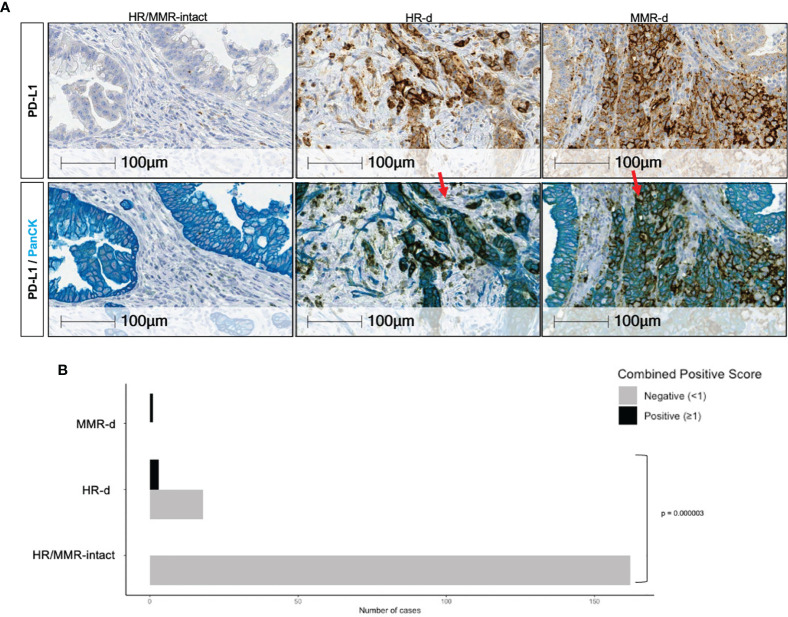
PD-L1 positivity in PDAC. **(A)** Representative PD-L1 immunostaining for HR/MMR- intact, HR-d and MMR-d PDAC. The top row shows tumors stained with PD-L1 (brown), while the bottom row shows the same tumor sections stained with PanCK (teal) following PD-L1 staining (brown). Red arrows in top panel show examples of PD-L1 staining. **(B)** Comparison of the proportion of cases in the HR/MMR-intact *versus* HR-d groups meeting the Combined Positivity Score (CPS) threshold of ≥ 1. Six of 25 HR-d cases had a CPS of ≥1, whereas none of the 163 evaluable HR/MMR-intact cases met the PD-L1 positivity threshold of ≥1. The MMR-d case scored >1.

We observed higher PD-L1 expression in the HR-d *versus* the HR/MMR-intact group (5.1 ± 11.9 *versus* 0.03 ± 0.1, p=0.0025). Six of the 25 HR-d tumors met the PD-L1 Combined Positive Score (CPS) threshold of ≥1, whereas none of the 163 evaluable HR/MMR-intact tumors reached the positivity threshold (p<0.00001; **Figures 3A, B**). The 6 HR-d cases with a CPS score ≥1 consisted of 4 cases with treatment-naïve primary PDAC biopsies (1024.001, 1183.001, 1235.001, 1337.001) and 2 metastatic biopsies (543.001, liver metastasis; 1099.001, peritoneal metastasis). Case 543.001 was treatment naïve at the time of biopsy, whereas case 1099.001 had undergone a course of neoadjuvant FOLFIRINOX for stage III disease. The biopsied metastatic peritoneal nodule was identified at the time of surgical exploration and the curative-intent resection was aborted following the intra-operative finding of metastatic disease. Importantly, the MMR-d case with treatment sensitivity to pembrolizumab also met the CPS threshold (**Figure 3B**, **Supplementary Figure 2**).

## Discussion

Genomic and transcriptomic profiling of PDAC has identified distinct molecular subclasses, such as HR-d (6). These advances have led to opportunities for targeted therapies and treatment stratification according to subclass assignment. Treatment responses to platinum-based therapies and PARP inhibitors have been demonstrated for HR-d PDAC (14, 27, 28). However, these therapies rarely lead to complete tumor responses and their durability is limited (17, 27). Therefore, there remains a clinical need to improve the current treatment strategies for HR-d PDAC. To this end, the genomic instability associated with HR-d PDAC may result in a tumor microenvironment amenable to ICI therapies. In the present study, we show increased intra-tumoral CD8+ T-lymphocyte infiltration and PD-L1 positivity by immunohistochemistry, corroborating previously reported transcriptomic evidence for T-cell inflammation in HR-d PDAC (22, 29). Although evaluation of the composition of cytotoxic, memory and exhausted intra-tumoral CD8+ T-lymphocyte populations in HR-d is subject to further investigation, there is evidence that infiltrating CD8+ T-lymphocytes are activated in HR-d. Indeed, Connor *et al.* showed that the cytolytic activity of infiltrating CD8+ T-lymphocytes, as measured by expression of granzyme A and perforin, and expression of co-regulatory genes, including cytotoxic T-lymphocyte antigen 4, programmed cell death 1 and indolamine 2,3-dioxygenase 1, was increased in HR-d and MMR-d PDAC (22). Moreover, these gene expression changes correlated with higher frequency of somatic mutations and tumor-specific neoantigens.

ICI therapies promote an anti-tumor immune response by disrupting signals that inhibit T-cell cytolytic activity (30). Cancers that exhibit immunogenicity, such as melanoma, have robust responses to ICI regimens with striking tumor regression and long-term survival (30). CD8+T-cell tumor infiltration has been associated with such potent and durable ICI treatment responses (31). Importantly, the proximity of cytolytic CD8 T-cells to tumor cell bed is predictive of ICI efficacy (32). However, while CD8+T-lymphocytes are adjacent to tumor cells in the microenvironment of melanoma tumors, the CD8+T-lymphocyte infiltrate in PDAC is primarily restricted to the stroma (33). Despite such observations suggesting that PDAC does not generally evoke an anti-tumor immune response, genomic and transcriptomic characterization of PDAC and its microenvironment has alluded to the existence of PDAC subclasses harboring immunogenicity (34, 35). The subclass prototype for PDAC with immunogenicity and responsiveness to ICIs is MMR-d, which exhibits elevated levels of tumor mutation burden (TMB) and tumor infiltrating CD8 T-lymphocytes compared to MMR-intact PDAC (19, 20, 36). Similarly, if the genomic instability that underlies HR-d PDAC leads to increased levels of neoantigens and tumor immunogenicity, the immune cell landscape of HR-d PDAC should also be distinguishable from the typical immune cold PDAC microenvironment. To this end, our results demonstrating elevated levels of intra-tumoral CD8+ T-cells in HR-d compared to HR/MMR-intact PDAC suggest that HR-d PDAC harbors a microenvironment with an inherent anti-tumor immune response. In support of the hypothesis that the genomic instability of HR-d tumors results in increased immunogenicity, a retrospective study of melanoma patients treated with ICI therapy found that tumors that regressed were enriched for *BRCA2* loss of function mutations and harbored a higher TMB (37).

We also evaluated the tumor microenvironment topography of FOXP3+ Tregs and CD68+ TAMs, since their immunosuppressive properties counterpoise the anti-tumor effect of cytotoxic CD8+ T-lymphocytes (38, 39). Tregs and TAMs may lower ICI efficacy and contribute to ICI resistance (40). Moreover, elevated levels of tumor infiltrating Tregs and TAMs correlate with poor outcomes in PDAC and other malignancies (31). We detected Tregs and TAMs in both HR-d and HR/MMR-intact cases, but we only observed a difference in the intra-tumoral density of Tregs between the two groups. The increase in intra-tumoral Tregs suggests an immunosuppressive counterbalance response to the anti-tumoral effect of the CD8+ T-cell infiltration. The MMR-d case with a durable response to pembrolizumab exhibited comparable levels of Tregs and TAMs in its intra-tumoral, peri-tumoral and stromal regions.

HR-d cases had a significantly higher CD8+:FOXP3+ ratio compared to HR/MMR-intact cases. The CD8+:FOXP3+ ratio may be a more functional measure of anti-tumor immunity and a better indicator of ICI responsiveness compared to CD8+ and FOXP3+ measurements alone (41). As the proportion of CD8+ to FOXP3+ rises, escape of CD8+ cytotoxic activity is facilitated with the dilution of Tregs and their inhibitory effects. The resultant milieu may also enhance the efficacy of ICI therapies. However, the CD8+:FOXP3+ threshold that signifies ICI responsiveness remains to be defined.

Although Tregs are considered to be pro-tumoral and have been associated with poor clinical outcomes in PDAC (31, 41), their role may be more complex. Depletion of Tregs in a transgenic mouse model of exocrine pancreatic cancer did not relieve the Treg immunosuppressive effects, but instead led to tumor progression (42). TAMs are also peculiar immune cells that exhibit functional plasticity with their phenotypic polarization governed by environmental signals (40). The functional continuum of TAMs ranges from pro-tumor to anti-tumor activity. In PDAC, immunosuppressive polarization of TAMs has been proposed, which is likely promoted by the predominantly hypoxic PDAC microenvironment (39). Although the exact contributions of Tregs and TAMs to the pro-tumor *versus* anti-tumor counterforces in the PDAC microenvironment are not fully understood, their roles are likely largely immunosuppressive considering their abundance across all PDAC and the generally immune cold PDAC microenvironment (31, 40, 43). Moreover, their prevalence in MMR-d may underlie the lower efficacy of ICIs in MMR-d PDAC compared to other MMR-d cancers (44). Therefore, ICI therapies PDAC subtypes with immunogenicity, including MMR-d, may be enhanced with the addition of agents that target suppressive immune cells such as TAMs (45).

Expression of PD-L1 by tumor or tumor-infiltrating immune cells has emerged as a clinical biomarker for anti-tumor immunity that may be enhanced with ICI therapy (23, 46). However, PD-L1 status is not a predictive biomarker of ICI sensitivity across all cancer types. Indeed, Davis et al. reviewed 45 FDA ICI approvals and found that PD-L1 was only predictive in 29% of cases, with the remainder either not tested or not predictive (47). Despite these limitations, in addition to MMR testing, PD-L1 expression currently remains the only feasible clinical assay for ICI treatment selection across cancer types (30). As such, we compared PD-L1 positivity by IHC in HR-d *versus* HR/MMR-intact PDAC, and found increased PD-L1 immunostaining in the HR-d group. Since guidelines for interpreting PD-L1 staining in PDAC have not been established, we applied the Combined Positive Score (CPS) that is recognized as a clinically relevant scoring method of PD-L1 positivity in solid tumors (23, 48). Although tumors that do not meet the CPS positivity threshold of ≥1 may respond to ICIs, tumors that reach the CPS cutoff have a higher likelihood of responding effectively to anti-PD-1 therapy. Strikingly, 6 of 25 HR-d cases and the MMR-d case met the ≥1 CPS threshold, while none of the 163 evaluable HR/MMR-intact cases reached the benchmark. These observations support a role for anti-PD-1 therapy in HR-d PDAC. Moreover, considering the heterogeneity of treatment responses to platinums and PARP inhibitors across HR-d PDAC (14, 17), sensitivity to anti-PD-1 may also be variable in HR-d PDAC and may be correlated with PD-L1 positivity. Finally, a limitation of this research registry-based study is the inclusion of cases that were treatment naïve at the time of tissue acquisition as well as cases following pre-treatment with chemo- and/or radiation therapy that may have altered their tumor microenvironment (49).

In summary, we demonstrate that HR-d PDAC exhibits a distinguishable tumor microenvironment with enhanced immunogenicity and PD-L1 positivity compared to HR/MMR-intact PDAC. These findings provide immunohistochemical correlation of the previously reported genomic and transcriptomic results classifying HR-d PDAC as a molecular subtype with inherent immunogenicity, and provide motivation for clinical trials to evaluate the efficacy of immunotherapy regimens in HR-d PDAC.

## Data Availability Statement

The original contributions presented in the study are included in the article/**Supplementary Material**. Further inquiries can be directed to the corresponding author.

## Ethics Statement

The study was conducted in accordance with the principles of the Declaration of Helsinki. The McGill University and the McGill University Health Centre (MUHC) Institutional Review Boards (#A02-M118-11A, #2018-3171, #2018-4139) approved the QPCS study, and the Institutional Review Board of the University Health Network (#15-9596) provided approval for the PanCuRx case series. All patients provided their written informed consent to participate in this study.

## Author Contributions

BGo: Conceptualization, Methodology, Software, Validation, Formal Analysis, Investigation, Data Curation, Writing – Original Draft, Writing – Review & Editing, Visualization YW: Conceptualization, Methodology, Validation, Investigation, Data Curation, Writing – Original Draft, Writing – Review & Editing, Visualization AT: Methodology, Formal Analysis, Investigation, Software, Writing – Review & Editing AP: Software, Data Curation, Writing – Review & Editing JR: Software, Writing – Review & Editing AC: Data Curation, Resources, Writing – Review & Editing CD: Data Curation, Resources, Writing – Review & Editing GBourd: Data Curation, Resources, Writing – Review & Editing RD: Software, Data Curation, Writing – Review & Editing GJ: Software, Data Curation, Writing – Review & Editing RG: Software, Data Curation, Writing – Review & Editing. AB Data Curation, Resources, Writing – Review & Editing BG Methodology, Validation, Resources, Writing – Review & Editing. AD: Methodology, Validation, Resources, Writing – Review & Editing JW: Methodology, Validation, Resources, Writing – Review & Editing GBourq: Software, Data Curation, Writing – Review & Editing GO’K: Methodology, Validation, Resources, Writing – Review & Editing SF: Methodology, Validation, Resources, Writing – Review & Editing CM-K: Resources, Validation, Writing – Review & Editing P-OF: Resources, Validation, Writing – Review & Editing AO: Resources, Validation, Writing – Review & Editing WF: Resources, Validation, Writing – Review & Editing SG: Resources, Conceptualization, Writing – Review & Editing M-CG: Resources, Validation, Writing – Review & Editing Z-HG: Resources, Validation, Writing – Review & Editing and GZ: Conceptualization, Resources, Writing – Original Draft, Writing – Review & Editing, Supervision, Project Administration.

## Funding

This work was supported by the Terry Fox Research Institute (project no. 1078), the Pancreatic Cancer Canada Foundation, the Quebec Cancer Consortium with funding from the Ministère de l’Économie et de l’Innovation du Québec through the Fonds d’accélération des collaborations en santé, and the PanCuRx Translational Research Initiative with funding by the Government of Ontario through the Ontario Institute for Cancer Research. Germline testing for the QPCS case series was partially subsidized by Invitae (San Francisco, CA). BGo was supported by a Canderel Graduate Studentship and the Cedars Cancer Institute Fellowship. YW was supported by a Vanier Canada Graduate Scholarship, the Fonds de recherche du Québec–Santé/Ministère de la Santé et des Services sociaux training program, and the McGill University Surgical-Scientist Program. JR was supported by the Fonds de recherche du Québec–Santé/Ministère de la Santé et des Services sociaux training program and the Hilton J. McKeown Stipend from McGill University. GZ is a clinical research scholar of the Fonds de recherche du Québec–Santé and recipient of the Michal & Renata Hornstein Career Award from McGill University.

## Conflict of Interest

P-OF has received honoraria from EMD Serono and consultation fees from Amgen, Bristol Myers Squibb, AstraZeneca Canada, Hoffmann La Roche, Merck Canada, Pfizer Canada and Roche Canada.

The remaining authors declare that the research was conducted in the absence of any commercial or financial relationships that could be construed as a potential conflict of interest.

## Publisher’s Note

All claims expressed in this article are solely those of the authors and do not necessarily represent those of their affiliated organizations, or those of the publisher, the editors and the reviewers. Any product that may be evaluated in this article, or claim that may be made by its manufacturer, is not guaranteed or endorsed by the publisher.

## Supplementary Material

Supplementary Figure 1 Kaplan-Meier survival curves for the HR-d and HR/MMR-intact groups.

Supplementary Figure 2 MMR-d PDAC. **(A)** IHC of QPCS case 750.001 showing intact nuclear MLH1 and PMS2 staining and absent nuclear MSH2 and MSH6 staining (red arrow) with intact stromal MSH2 and MSH6 staining (blue arrow) to indicate MMR-d. **(B)** Computed tomography showing mesenteric recurrence (orange arrow) following surgical resection of the primary. **(C)** Computed tomography following 18 months of pembrolizumab treatment showing a decrease in the mesenteric recurrence (orange arrow), indicating partial treatment response.
