# Supplementary figures and images for: Intra-Tumoral CD8+ T-Cell Infiltration and PD-L1 Positivity in Homologous Recombination Deficient Pancreatic Ductal Adenocarcinoma
Source: Front Oncol. 2022 Apr 25;12:860767. doi: 10.3389/fonc.2022.860767 (PMC9082359; doi:10.3389/fonc.2022.860767)

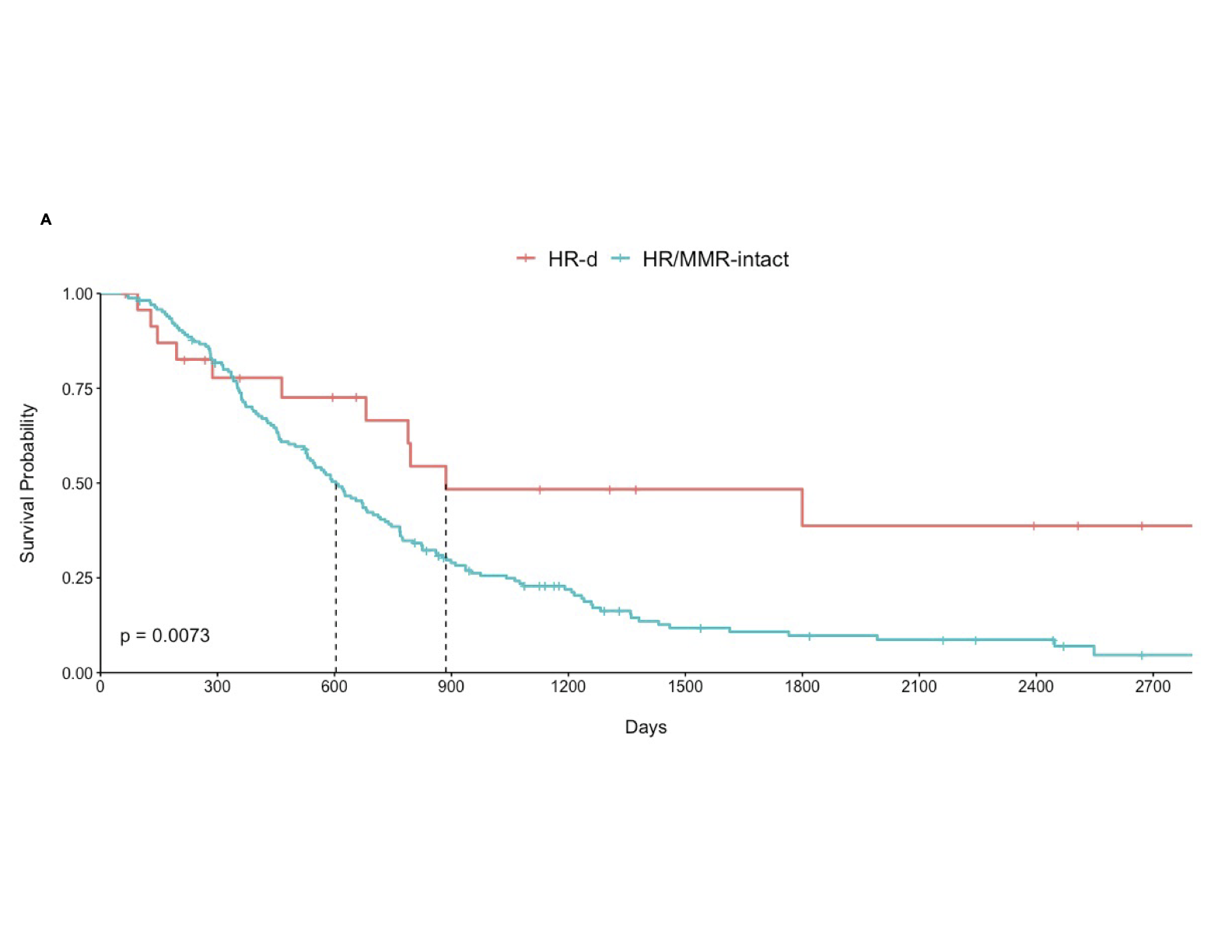

Supplement: Supplementary Figure 1 — Kaplan-Meier survival curves for the HR-d and HR/MMR-intact groups. [file Image_1.tiff]

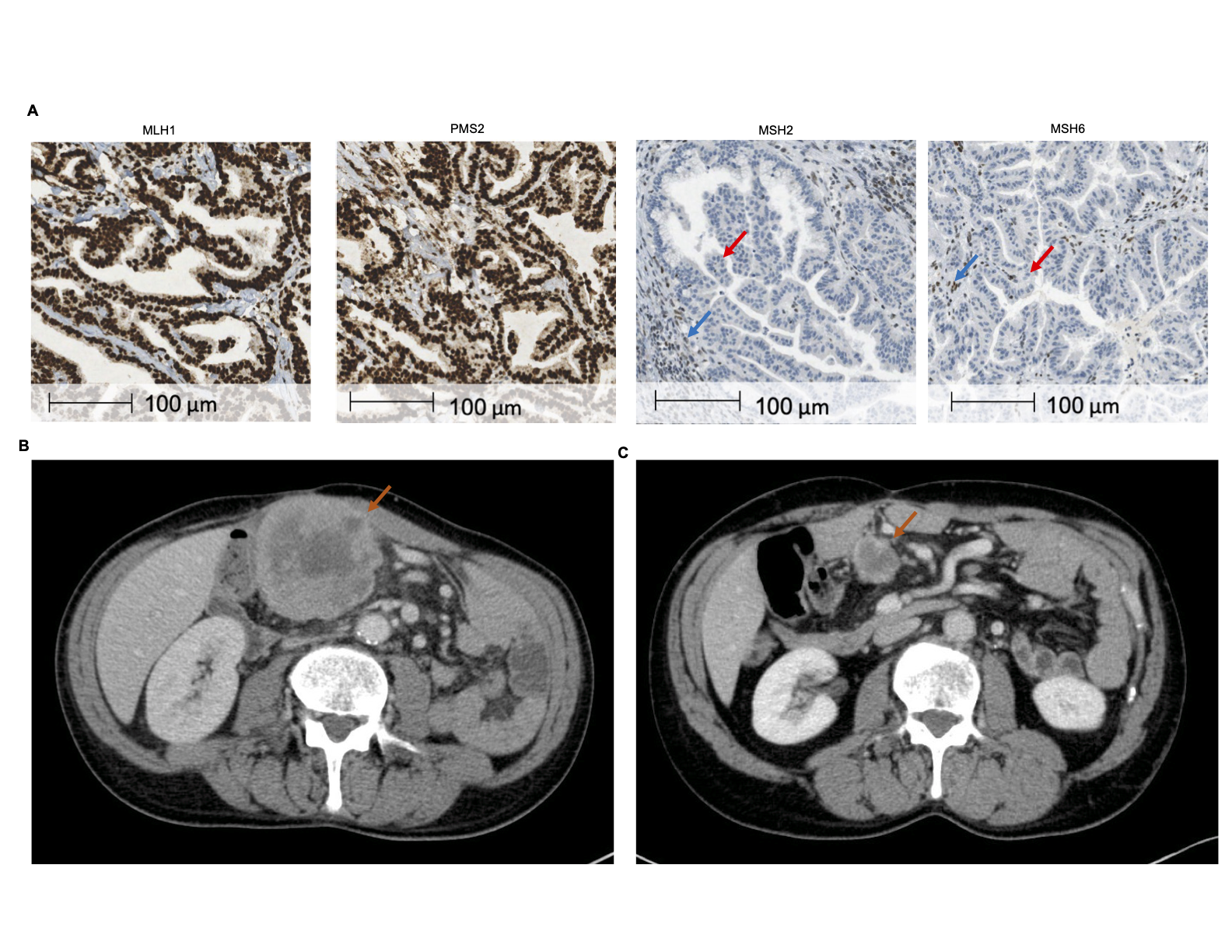

Supplement: Supplementary Figure 2 — MMR-d PDAC. (A) IHC of QPCS case 750.001 showing intact nuclear MLH1 and PMS2 staining and absent nuclear MSH2 and MSH6 staining (red arrow) with intact stromal MSH2 and MSH6 staining (blue arrow) to indicate MMR-d. (B) Computed tomography showing mesenteric recurrence (orange arrow) following surgical resection of the primary. (C) Computed tomography following 18 months of pembrolizumab treatment showing a decrease in the mesenteric recurrence (orange arrow), indicating partial treatment response. [file Image_2.tiff]
